# A Medical Causerie

**Published:** 1916-07-01

**Authors:** 


### A MEDICAL CAUSERIE.

The hope that the union of the various medical societies in the Royal Society of Medicine would avoid the necessity of new organisations has to a very large extent been realised. But the centrifugal tendency has not quite disappeared. A year or two ago some gentlemen interested in dermatology set up a new society in this department, and judging by the frequency with which the same names recur in the published accounts of the proceedings their early zeal has not abated. Now we have a new development in the study of diseases of the ear and throat, and it calls itself the Oto-Laryngological Society. It must be regarded as a rival to the sections of Otology and of Laryngology of the Royal Society of Medicine. Thus the position to-day reverts very much to what existed until some ten years ago, when there was very acute rivalry between two societies concerned with disease of the upper air-passages. Each had its champion or protagonist and there were occasionally very lively passages. Not a few critics of the Royal Society's scheme believed that union would mean lethargy due to the absence of stimulation and competition, but the event has largely dispelled this fear. Still it is evident that a few men continue to lean towards isolation and detachment, or, it may be, that not receiving what they consider proper recognition on the orthodox platform they decide to set up a conventicle of their own. The Fellows of the new Oto-Laryngological Society have put on record the announcement that "they did not think it possible" that members of the medical profession could have acted as the German doctors are said to have done at Wittenberg Camp; also "they are not a little surprised" that medical opinion on this behaviour has not yet made itself heard in this or in neutral countries.

A correspondent writing in the *Lancet* quotes an experience which is of interest in relation to the proposed cultivation of medicinal plants in this country and which shows that the following of agricultural pursuits is by no means incompatible with a smart business method. One of the leading drug firms used to receive every year from a farm labourer a considerable parcel of hemlock seed for which a price some 50 per cent. below the market quotation was asked. When the vendor was requested to explain the position he replied: "Early in the year I fill my pocket with seed and go out into the country. Whenever I come across a good wide hedgerow I sow the seed all along it. Later in the year, when the plants have grown and seed has formed, I again visit the hedges, call the attention of the farmers to the noxious weed, and offer to cut it away at the modest price of one shilling a hedge. So you see I get my land for nothing and, in addition, get paid for cutting my own harvest." The ingenuous agriculturist deserves congratulation. His rival would seem to be a man who having discovered a cure for the itch arranged with a companion that this latter should acquire the disease and should then travel through the countryside shaking hands with as many people as possible. The original discoverer followed at an interval of some ten days and provided, on payment duly made, the antidote for the bane which his colleague had effectively distributed; the partnership is an illustration of a union of scientific knowledge and business enterprise.

All his colleagues and his numerous friends have congratulated Mr. James Berry on his safe return to London after his adventurous experiences in Serbia and his capture as a prisoner of war by the Austrians and Hungarians. In this latter direction the party were much more fortunate than some of those who have fallen into the hands of the Germans. Mr. and Mrs. Berry have written an account of the work which they were able to carry out, and this will shortly be published under the title of "The Story of a Red Cross Unit in Serbia." The book will give a general account of how hospital, sanitary, dietetic, and personal difficulties were surmounted with the aid of limited material but abundance of good temper. Peace does not always reign in these missionary enterprises, and in more than one hospital staff events have brought out very acute contention. Such things have happened even among the gentler sex! Another medical man who has had moving experiences in Serbia, and who also is now back again in London, is Dr. R. O. Moon. He embodied some of his recent experiences in the Near East in his recent Chadwick lectures.

The late Sir Frederic Goodhart had a decided objection to the application to himself of the term "specialist." It is said that his objection took a pronounced form when a lady bringing her little daughter to him announced that she had taken this step because she understood he was a "specialist for children between six and seven years of age"! There is another consulting physician who entertains a similar view, and it is said that on one occasion, as he answered his telephone, a sharp American voice inquired, "Are you the doctor?" to receive the reply, "Well, madam, I am *a* doctor." Question number two was "Are you a baby specialist?" and here there was even less delay in the response, "No, madam, I am a full-grown man!" Considering how the word "specialist" is used in Bond Street and the neighbourhood it is astonishing that the medical profession so often encourages it within its own ranks. In some measure the public is responsible for its introduction as a medical term, but this merely means that doctors do not take the trouble to explain to their patients the exact situation. One result is that certain persons who are entirely innocent of all medical training are often regarded as qualified practitioners because they describe themselves as "specialists." "Physician" or "surgeon" they dare not use, but "specialist" is at the disposal of any quack-salver.

